# Efficacy of a family practice-based lifestyle intervention program to increase physical activity and reduce clinical and physiological markers of vascular health in patients with high normal blood pressure and/or high normal blood glucose (SNAC): study protocol for a randomized controlled trial

**DOI:** 10.1186/1745-6215-12-45

**Published:** 2011-02-16

**Authors:** Robert J Petrella, Kuni Aizawa, Kevin Shoemaker, Tom Overend, Len Piche, Mauricio Marin, Sheree Shapiro, Sophie Atkin

**Affiliations:** 1 Schulich School of Medicine, Dept of Family Medicine, University of Western Ontario, Canada; 2 Schools of Kinesiology, Faculty of Health Science, University of Western Ontario, Canada; 3 Physical Therapy, Faculty of Health Science, University of Western Ontario, Canada; 4 Brescia College, Division of Food and Nutritional Sciences, University of Western Ontario, Canada; 5 Diabetes & Vascular Medicine Research Centre, Peninsula Medical School, University of Exeter, Exeter, UK; 6 Lawson Health Research Center, London, N6C 5J1, Canada

## Abstract

**Background:**

Previous interventions to increase physical activity and reduce cardiovascular risk factors have been targeted at individuals with established disease; less attention has been given to intervention among individuals with high risk for disease nor has there been determination of the influence of setting in which the intervention is provided. In particular, family practice represents an ideal setting for the provision and long-term maintenance of lifestyle interventions for patients at risk (ie high-normal blood pressure or impaired glucose tolerance).

**Methods/design:**

The Staged Nutrition and Activity Counseling (SNAC) study is a randomized clustered design clinical trial that will investigate the effectiveness and efficacy of a multi-component lifestyle intervention on cardiovascular disease risk factors and vascular function in patients at risk in primary care. Patients will be randomized by practice to either a standard of care lifestyle intervention or a behaviourally-based, matched prescriptive physical activity and diet change program. The primary goal is to increase physical activity and improve dietary intake according to Canada's Guides to Physical Activity Healthy Eating over 24 months. The primary intention to treat analysis will compare behavioral, physiological and metabolic outcomes at 6, 12 and 24 months post-randomization including estimation of incident hypertension and/or diabetes.

**Discussion:**

The design features of our trial, and the practical problems (and solutions) associated with implementing these design features, particularly those that result in potential delay between recruitment, baseline data collection, randomization, intervention, and assessment will be discussed. Results of the SNAC trial will provide scientific rationale for the implementation of this lifestyle intervention in primary care.

**Trial registration:**

ISRCTN: ISRCTN:42921300

## Background

The epidemic of sedentary lifestyle and poor eating habits, with associated escalation of cardiovascular disease and risk factors, has challenged researchers to develop new strategies aimed at preventing these growing health problems [1]. Efforts to prevent the morbidity and mortality of these chronic diseases as a result of poor lifestyle have focused mainly on the clinical management of individuals with existing disease. Such an approach does not address the potential reversible causes of these conditions, primarily poor lifestyles including low levels of physical activity and poor dietary habits.

Elevated blood pressure and elevated blood glucose are prototypic of preventable chronic cardiovascular disease risk factors [2]. Both are common conditions representing approximately 5-25% of the adult population. However, many more individuals are at risk [3]. For example, there is an estimated 90% lifetime risk for the development of hypertension among those with high-normal blood pressure [3]. Recent evidence has shown the importance of lifestyle interventions for the prevention of type-II diabetes amongst individuals with impaired glucose tolerance, and high-normal blood pressure and hypertension [4-7]. Hence, the unprecedented epidemic of cardiovascular risk amongst Canadians which parallels a growing aged, sedentary, and overweight population, has occurred despite the unequivocal evidence supporting lifestyle for the prevention of cardiovascular disease. The result is an urgent need for the investigation and delivery of evidence-based strategies to improve lifestyle behaviour resulting in acute and sustainable changes in cardiovascular function and health. To have maximal impact in preventing further disability, we will target those patients with preclinical risk for cardiovascular morbidity (namely those with high normal blood pressure and/or impaired glucose tolerance). The catalyst for this bench-to-bedside model will be the family physician, who will deliver this research intervention in his/her practice to a representative population of adults at risk.

### Rationale for our intervention

The Framingham Heart Study found that individuals with high normal blood pressure were 2-3 times more likely to regress to hypertension than those with optimal blood pressure [3]. In fact, 37% of individuals with high normal blood pressure progress to hypertension over a four-year follow up. Particularly disturbing, in terms of prevention, is the trend for progression to hypertension being worse over the last 20 years compared to the period of 1952-1975 suggesting that an aging, more obese and sedentary population is placing the burden at epidemic levels. Epidemiological research in type 2 diabetes (T2D) shows a relative risk of death from cardiovascular causes compared to non-diabetic age-matched control ranges from 1.5 to 2.5 higher in men and 1.7 to four times higher in women [8]. Further, the rates of cardiovascular morbidity in diabetics can even be higher and exacerbated by increasing age where impact can be at 20 times greater risk of coronary heart disease compared to non-diabetic controls [9,10]. The risk of coronary heart disease events has increased even in newly diagnosed T2D, probably due to latent period of impaired glucose tolerance during which atherogenic effects, including endothelial dysfunction, contribute to a "ticking clock" phenomenon. The epidemic of T2D parallels hypertension prevalence being clustered in an aging, obese, and sedentary population and the coexistence of hypertension, impaired glucose tolerance and diabetes have devastating consequences despite the unequivocal evidence supporting the impact of lifestyle interventions. Indeed, recent studies have shown that lifestyle intervention alone, or even compared to glucose modifying medications, is effective in preventing the development of type-II diabetes in those with impaired glucose tolerance [4,5]. Further, physical activity and diet have been shown effective in treating hypertension and T2D including amelioration of vascular to end-organ changes [11]. These population trends in the face of growing preventive evidence suggest there is an urgent need for the implementation of preventive lifestyle strategies to impact the epidemic of cardiovascular risk [12]. The approach of waiting for hypertension or T2D to develop and only then to treat pharmacologically is contrary to best evidence, best practice, and is injudicious. Stammler noted that current practice in prevention is late, defensive, reactive, time-consuming, associated with side effects, and noncompliance, costly, only partially successful and endless [13].

Given the high burden of cardiovascular disease in the population, where is intervention targeted? Family practice is the ideal setting for a lifestyle intervention, particularly in the long-term, since there is a continuity of care required for long-term success, there is promotion and complimentary preventive health strategies for cardiovascular health in place. There is also great potential for interaction with a large population at risk, and patients identify their family doctor as a preferred source of lifestyle information [14]. The Canadian health care system and family practice, in particular, provide the ideal framework through which to implement lifestyle changes among patients at risk since family physicians are the point-of-entry and provide continuity within the health care system.

With family physicians as a primary source of health information, prevention and promotion, and an important point of contact and influence for Canadians, it is paradoxical that for the most part, family physicians do not counsel or monitor lifestyle habits [15,16]. In fact, family physicians believe in the merits of increased physical activity and approving diet among their patients, yet very few counsel or provide written instruction for their patients regarding the support and health behaviour [17,18]. The reasons for this discrepancy are many and include lack of time, training, and tools [19]. A succinct message and strategy from the family physician could be a potent catalyst to motivate changes and lifestyle habits among patients. However, the message in terms of lifestyle intervention in primary care has been sparsely investigated to date and, specifically, has not been addressed among those at high risk for hypertension and T2D [15,20].

Since 1993, the U.S. and Joint National Committee on the detection, evaluation, and treatment of high blood pressure and the working group report on primary prevention of high blood pressure have recommended lifestyle modifications for blood pressure control, including weight loss if overweight, sodium reduction, increased physical activity, and limited alcohol consumption [21,22]. Similar recommendations have been made for T2D [23]. Based on the results of dietary approaches to stop hypertension, the 7^th ^Report of the Joint National Committee and the recent Canadian Hypertension Education Program have also specifically recommended the DASH diet, which includes a diet rich in fruits, vegetables, and low fat dairy products with an emphasis on reduced overall saturated fat and total fact and cholesterol content [24-26]. While the DASH diet has been shown effective in reducing blood pressure, little is known about the effectiveness of simultaneously implementing different lifestyle recommendations at the same time (i.e. physical activity and a health diet) nor has the impact of this and other lifestyle interventions when implemented at the level of primary care been described [20]. Indeed, most trials that have studied the effects of lifestyle interventions on blood pressure and blood glucose have tested the impact of just one lifestyle change in isolation and few trials have tested the effects of multi-component interventions simultaneously [27,28].

Since recommendations support lifestyle and some have gone as far as to endorse specific program interventions, it is notable that no trial has tested the impact of these interventions and free-living persons selecting their own diet or physical activities nor have they undergone the rigor of testing in the primary care setting [24]. Studies done in primary care could test or trigger dissemination and implementation more globally among larger populations at risk.

### Challenges with the intervention

Behaviour staging using the principles of the transtheoretical model has not been considered in many lifestyle intervention trials [29]. This in turn may have had impact on the long-term sustainability of changes that were observed. Previous lifestyle trials have also not consistently standardized the measurement or prescription of physical activity or dietary behavior [27,30-34]. Further, there has also been a lack of matching of physical activity to other lifestyle habits, such as dietary intake. Finally, whether the observed changes in behaviour and clinical outcomes are related to changes in basic physiological mechanisms that control health is also essential to understanding the impact of these interventions on the pathophysiology of hypertension and T2D.

In this setting of an epidemic of cardiovascular disease and poor lifestyle, the overall aim of the Staged Nutrition and Activity Counseling trial (SNAC) is to determine the blood pressure and blood glucose lowering effects of a matched or scaled physical activity and dietary lifestyle intervention among persons with high normal blood pressure and/or impaired glucose tolerance. Further, as most of these patients are seen in family practice, and given the key role family physicians may provide in achieving and sustaining lifestyle change, the intervention will be delivered compared to the usual care of family practice lifestyle management. Finally, few studies have suggested that lifestyle interventions (primarily single interventions) can improve physiological determinants of cardiovascular function and health [35,36]. Whether this is similar in a large primary care intervention with two simultaneous lifestyle interventions is unknown. It is anticipated that the results of SNAC will provide scientific rationale and guidance for the implementation of this important lifestyle intervention program designed to prevent hypertension and T2D among those at risk for cardiovascular disease.

## Methods/Design

The delivery of the SNAC protocol within the family practice unit using a brief 10-15 minute intervention geared to fit within a normal clinic session is critical to the potential implementation of the intervention in family practice. This follows previous developmental work with the STEP intervention which was found to be effective and acceptable to family physicians [19,37]. Training for the intervention will include a 30-minute workshop that promoted a team approach to identification of patients and delivery of the intervention by the family physician. The unit of analysis in SNAC is the individual patient, while the unit of randomization is the practice itself. Hence, it was deemed necessary to consider a cluster randomized, controlled design to determine the effectiveness of SNAC. Both STEP and SNAC have been endorsed by the College of Family Physicians of Canada.

The aim of this paper is to describe the design, features, and practicalities of the trial that may be common to other cluster randomized trials within a primary care setting.

### Design

SNAC is a multicentre, cluster-randomized trial. Participating sites will include 20 clinical centres (6 academic family practices and remaining 14 non-academic centres). The Heart, Health, and Exercise Laboratory at the University of Western Ontario will serve as the central laboratory for all physiological and biochemical collections, and analyses. Recruitment of all clinical sites has been previously described with a catchment of over 150,000 patients [38]. The diet assessment and composition of the Mediterranean diet choices will be coordinated through Brescia College School of Nutrition at the University of Western Ontario, including quantification of dietary consumption using Food Processor (v 1.0). Serum measurement of free fatty acid levels (under the supervision of Dr Bruce Holub, University of Guelph) and measurement of endothelial markers of vascular function will be processed in the lipid analysis laboratory at McMaster University. Plasma leptin levels will be measured in the laboratory of Dr. Peter Lemon at the Exercise Nutrition Laboratory at the University of Western Ontario. The study has been funded by the Heart and Stroke Foundation of Canada and was approved by the Institutional Ethics Review Board of the University of Western Ontario.

### Study Population

Primary care practices will be recruited using a family practice research network. Randomization to intervention or usual care control will be done by a random number table. Recruitment of patients within the practices will include two successful strategies previously utilized by our group including opportunistic recruitment of consecutive patients during the course of routine, prescheduled, clinic visits or query of the network database for inclusion criteria and screening of patient visit lists [19,37]. All practices will target 10-12 patients each over a 6-month period.

#### Intervention under trial

##### Content of intervention

We based the content of the intervention on recommendations for dietary and physical activity lifestyle management made by several systematic reviews and recommendation processes [23,24,26,39,40]. These were supplemented by our own experience and success using the STEP program for physical activity adoption among primary care physicians [37]. Further refinement has included the piloting of the SNAC diet activity prescription among representative groups of patients [41]. Resource materials and training for those in the intervention group will be facilitated by dieticians including the development and delivery of grocery tours, cooking demonstrations, as well as meal preparation and menus according to Mediterranean style diet [42]. A training package for physicians has been prepared to assist in standardizing the implementation of SNAC materials in their practice. A workshop will provide skills and behaviour staging in diet and physical activity counseling and prescription. Patients will be staged separately for both physical activity and dietary change readiness and then asked to perform an exercise step test to determine exercise training heart rate and activity diet prescription for each patient [43]. Determination of fitness within each subject will be translated into a low, moderate, or high category when compared to population averages and this will be translated into the prescription of specific consumption of recommended fruits, vegetables, grains, meat products, and using a Mediterranean diet algorithm [44]. A selection of resource materials matched for stage of readiness in terms of their content for increasing physical activity levels, as well as adopting the Mediterranean diet by subjects will be provided to participating physicians and staff during a workshop. Key items for review at each study visit will be highlighted to promote adherence and to prevent drop out during the clinical trial period. Patients will be eligible if they have had high normal blood pressure determined as a seating resting systolic blood pressure between 130 and 139 mmHg and/or a diastolic blood pressure of 85-90 mmHg based on average of three repeated blood pressures, and impaired glucose tolerance determined as a fasting glucose level between 6 and 6.9 mmol/l plus a 2 hour post 75 glucose challenge of 8-11 mmol per litre. Patients with hypertension or T2D will be excluded. Other inclusion criteria will include age from 30-85 years, willingness to participate in the interventions and participation over a 12-month period and the presence of High-normal blood pressure or IGT/IFG. Exclusion criteria will include: Myocardial infarction within 3 months prior to study; Coronary artery bypass within 3 months prior to study; Cerebrovascular ischemia/stroke within 3 months of study (including TIA); Previous (>6 months) diagnosis of hypertension or T2D; Angioplasty within 3 months of study; Symptomatic congestive heart failure; Atrial flutter, presence of controlled or uncontrolled hypertension: Unstable angina: Unstable pulmonary disease (e.g. asthma or obstructive lung disease); Use of medications know to affect heart rate (e.g. β-blockers); Second or third degree heart block, History of alcoholism, drug abuse, or other emotional, cognitive or psychiatric problems that are likely to limit compliance to the study; Pacemaker; Unstable metabolic disease (e.g. uncontrolled diabetes glucose >15mmol at screening); Orthopedic or rheumatologic disease (i.e. severe rheumatoid arthritis); Known Autonomic Nervous System Disorders such as Raynaud's Syndrome or Autonomic failure; Already enrolled in clinical research trial.

### Randomization

Eligible practices will be randomly assigned to either SNAC or usual care control by a computer program. All practices in the intervention group will attend a training session as described above, while the usual care group will attend a sham hypertension guidelines training program. Both training sessions will be matched for duration.

Eligible participants who provide informed consent will attend a baseline visit for data collection. At this visit, patients will be prescribed a lifestyle program either SNAC or usual care. In the SNAC intervention, patients will be staged and the activity:diet prescription will be delivered. Those in SNAC will receive stage-matched resources, schedules for cooking demonstrations and grocery tours, and consultation appointment cards for study kinesiologists or dieticians. Both groups will be given study visit schedules. The visit schedule is provided in Table 1.

**Table 1:** Schedule of Observation and Procedures

Assessment	Screening	Baseline	Week 8	Week 16	Week 24	Week 52 Final Visit
Informed Consent	X					

Medical history	X					

Physical exam	X					

Randomization		X				

Clinical Blood Pressure		X	X	X	X	X

24 hour ambulatory blood pressures		X	X	X	X	X

Fasting glucose/blood profile		X				

2 hour glucose tolerance test		X				

Physical Fitness		X	X	X		X

Behavioural Measures		X	X	X		X

Physiologic Measures		X	X	X		X

Drug			X	X		X

Placebo			X			

### Blinding

Clinical centre staff at the Heart, Health and Exercise Laboratory will be blinded throughout the trial.

### Interventions

The theoretical basis for the SNAC intervention is modeled within the social cognitive theory and transtheoretical stages of change. This approach emphasizes the importance of an individual's ability to regulate their own diet and activity behaviour by setting goals, monitoring progress towards these goals, and attaining the necessary skills to reach these goals. These approaches also seek to increase the self-efficacy and outcome expectancies both of which are critical mediators of behaviour change. The transtheoretical model also recognizes that behaviour change is a dynamic process. Different behaviour strategies may be emphasized depending on the individual stage of readiness for change. In addition, the SNAC intervention also emphasizes motivational counseling techniques. Input from all members of the family practice team including kinesiologists and dieticians will be enlisted as would be the case in usual care practice. Further, behaviour change will be targeted through a tailored diet:activity prescription. Hence, the theoretical basis for SNAC contrasts sharply with the delivery of lifestyle modifications in the usual clinic settings, which rely primarily on verbal advice without attention to specific diet:activity targets within individuals nor do they attend to behaviour change or referral to allied health specialists [15,16]. This would support the approach patients would prefer and what best evidence suggests is most effective [16,17].

### Schedule of visits

After initial baseline assessments, patients will be counseled to adhere to their new physical activity and dietary recommendations over the next three months. Follow up in this 3 month block includes unencumbered access to the study physician as well as to a dietician and/or kinesiologist in the coordinating centre. To facilitate adoption of a Mediterranean diet, one meal per day (5 days per week) will be offered to patients by the University of Western Ontario Food Services Program or for the first eight weeks of the program. This will serve to familiarize the subjects with the quantity but also the quality of the Mediterranean diet. Since this approach does not provide all meals, we do not believe this would influence our clinical or physiological outcomes in the longer term. Patients will be advised to collect 3-day food records prior to their next visit as well as to log their physical activity behaviours. At each 3-month interval, the patients will return to the family practice to have repeat measures done including diet:activity restaging and a new diet:activity prescription. This will be followed by laboratory assessments of the physiological, biochemical, and anthropometric measures. The family physician visit also offers the opportunity to reinforce activity dietary change.

### Study Measurements

Measurements will occur at baseline, 2-months, 6-months, 9-months, 12-months and 24-months post intervention (Table 1). Primary and secondary outcome measures are described as clinical, behavioral and physiological.

### Primary and Secondary Outcome Measures

#### Primary Outcome Measures

a) clinical - clinic blood pressure with an automated BP device BPTRU™; fasting glucose, b) physical fitness - predicted VO_2 max _using STEP test and measured VO_2 max _using a modified Balke exercise treadmill protocol; BMI, c) physiological measures: brachial and carotid artery endothelial flow-mediated dilation and function.

#### Secondary Outcome Measures

a) clinical - 24-hour ambulatory blood pressure; 2-hour glucose tolerance testing, hemoglobin A1C, microalbumin; waist circumference, calorie counts, b) behavioural measures - stage of change, self efficacy for diet and physical activity change, quality of life (SF36), barriers and benefits scales, c) physiological measures - waist circumference, calorie counts, dietary composition, cardiac function (echocardiographic determination of systolic and diastolic function, and left ventricular wall dimensions and volume), vascular geometry and elastic properties of the carotid and brachial arteries, muscle sympathetic and nerve activity discharge patterns at rest and a response to lower body negative pressure. Blood chemistry included blood lipid profiles homocystine, serum insulin, rennin, aldosterone, cortisol, C-reactive protein (CRP), markers of endothelial function including vascular adhesion molecule (VCAM) and intracellular cell adhesion molecule 1 (ICAM-1), urinary catecholamines, and serum free fatty acid composition.

Effects dependent on demographic subgroups will be defined by age, gender, and practice setting. Since previous trials have suggested that lifestyle interventions achieve their greatest effects early on, and that effects tended to diminish over time, we will evaluate the durability of these effects to 12 months and then for a further 12 months thereafter to determine the intervention rate of decay.

### Sample Size and Data Analysis

#### Sample Size

Data from a previous "feeding" trials including the DASH trial have estimated a standard deviation change in systolic blood pressure after the intervention was between 5.32 and 5.92 mmHg providing a 90% power [24]. In terms of change and blood glucose levels, 0.1 to 0.3 mmol. reduction are considered important in differentiation of the risk for the development of T2D [4]. For physiological outcomes' sample size estimate, we chose a mean difference between groups of 0.25 mm standard deviation as appropriate for change in vascular dimension. Previous work in our laboratory support the ability to detect the 2 mm difference in vessel diameter with a standard deviation of .025 mm following a similar lifestyle intervention. Hence, we required at least 10 subjects per group (two-tailed t-test, p < 0.025, power = 0.8). Assuming an 80% success rate in achieving suitable recordings in two test sites and a drop out rate of approximately 20%, we will recruit 40 subjects per treatment arm to the high normal blood pressure and IGT groups.

Initially, we anticipated recruiting about 5-10 patients per practice using a cluster design. To allow for the effect of this clustering we used an interpractice correlation coefficient (ICC) of 0.025. We felt this would be a conservative upper limit for the ICC on the basis of previous work in primary care. The inflation factor was therefore calculated to be 1+0.025 (10 minus 1) = 1.05. This figure gives a required number of patients per treatment arm of approximately 90 equivalent to 10 practices recruiting about 10 patients each.

However, there is some theoretical loss of power from using variable cluster sizes. This is negligible in the current study of approximately 50 patients per treatment arm. Hence, our allocation method of 10 patients per practice was designed to avoid this possibility. Statistical analysis will include differences among the subjects' characteristics in clinical, behaivoural, and physiological outcome measures using analysis of variance (ANOVA). Interactions and/or a specific age, gender, or clinical variable differences will be examined using Tukey's post hoc analyses. If a group difference in a potentially confounding variable is observed at limit differences in a key outcome, analysis of covariance (ANCOVA) will be performed with the potentially confounding variable serving as the covariant. Relations of interest will be initially identified by univariate correlation analysis. Independent correlations among the dependent variables will be determined using partial correlation analyses and/or conventional multivariate step-wise regression analysis. In all cases, probability levels will be p < 0.05.

### Delivery of Lifestyle

Practical issues arise in the delivery of lifestyle interventions in primary care. Previous pilot work by our group has identified contamination of controlled practices could occur, despite using a cluster randomized design. However, this contamination has been small and would favour the null hypothesis. Contaminations can include non-study related public education or public health/physicians targeting dietary and lifestyle interventions that occur as "background noise" intended for use in various clinical settings. We have partnered with local public health and pharmaceutical industry to minimize the impact of these contaminations. Contamination could also arise from patients moving between clinical practices. This will be negligible in the current trial as all clinic staff and patients are rostered to the study practice and cross-coverage between practices is not encountered. These potential contaminants will be discussed during the SNAC and control training sessions for study physicians and staff.

### Timing of the Intervention

Pilot work in our lab has demonstrated that it is impractical to impose strict study start times and recruitment targets within some practices. Hence, there will be no particular matching of start times or recruitment activity between practices. Support will be given to all practices to increase or maintain their recruiting activities over the first two months of their involvement in the trial and given the demands of the clinical settings, all practices will be encouraged to complete recruitment for the trial within three months of their enrollment and training. Our previous work supports anticipated success of this strategy.

### Timing of Recruitment

A key design feature of this trial is that the allocation of practices within the research network will be carried out prior to the recruitment of the required number of patients. This will be done to ensure proper understanding of the trial and review recruitment strategies to avoid selection bias utilized by our group in a previous randomized controlled cluster design of physical exercise prescription in primary care [45]. Similar differences in patient characteristics at baseline between the SNAC and the control group in this trial allow for valid interpretation of results. We do not anticipate a difference in recruitment times between the two groups because both groups will be counseled regarding recruitment strategies for the trial at the same time. This may be in contrast to other studies where slow uptake of control practices have been reported should the treatment allocation be known prior to recruitment. Hence, practices would potentially see little gain from their participation. Further, unequal timeframes for recruitment of patients to the intervention or control groups could bias comparison. For example, there is good evidence for seasonal variation on the lifestyle factors of physical activity and diet. If either of the groups recruited more patients during a particular season or it was stratified to do so, then they could be expected to have a change in one of the lifestyle interventions regardless of the intervention itself.

### Recruitment of Practices

This trial was designed to include practices in Southwestern Ontario, which has a higher burden of cardiovascular disease than other parts of the province [44]. We have aimed to recruit a total of 20 practices to the trial with stratification for urban, rural, and academic practice affiliation distribution. We then would obtain a representative balance of practice settings within the region.

### Recruitment of Patients

It will be the responsibility of each participating practice and not the research coordinating centre staff to recruit consecutively attending patients with high normal blood pressure and/or impaired glucose tolerance within the selection criteria. Any member of the primary care team can recruit patients. It is anticipated the process would be completed within 2-3 months of enrolment into the program. Delay in recruitment of some practices, due to unforeseen changes in the practice environment including staff leave, vacations, and competing interest in other clinical trials, will be minimized given the established activity of the research network. For example, once a practice has recruited its quota of patients, the strategy used to achieve this quota will be shared with those in the same intervention group by the research staff. No incentives will be utilized as this will not be the case in usual practice. It is apparent that some patients, given the nature of the intervention and assessments, will drop out before the baseline data collection. If this recurs, every effort will be made to replace the patient prior to the randomization. In addition, there is a possibility that some patients will have an exacerbation of their blood pressure or blood glucose through the randomization phase requiring other forms of treatment. This will be treated as an exclusion for the study and patients will be withdrawn. This will not lead to a bias in comparison to the intervention and control arms as both groups will be subjected to the same risk. Whether this could influence the generalizability of the intervention and control situations to general practices is also not an issue as many patients with high normal blood pressure or impaired glucose tolerance will convert to hypertension or T2D at any point in time. Record of those who are withdrawn from the protocol at any point will be analyzed to identify other aspects of prevalence of these conditions and determinants of adherence within the family practice settings.

The non-random selection of practice patients within the study practices could have impact on the generalizability of the results of this trial. However, this is standard in most randomized clinical trials, whereby convention, patients in participating centres are selected systematically rather than randomly. Implementation of the findings of randomized, controlled trials is an important area of research by our group. Importantly, practices in this trial will be balanced for a practice setting (academic, rural, urban) and will enhance the generalizability of the communities they represent in the future implementation of dissemination strategies.

### Treatment allocations

#### Timing

In an ideal world, patients in this trial would be recruited, provide consent, and physicians/researchers would obtain a baseline clinical data collection in the morning followed by laboratory assessments within the next 24 hours for completion of the clinical, behavioural, and physiological measures. In a trial such as ours in primary care, however, each of these stages takes a variable amount of time, planning, and scheduling within each practice. We have designed the trial in a way to ensure that each stage takes place as quickly as possible after the previous stage (within one week) so as to avoid the potential problems of patient drop out or change in physiological and clinical variables over a longer period of time.

Recruited patients will be consented in their practice setting while baseline clinical, behavioural and physiological measures will be collected in the Heart, Health & Exercise Laboratory. Patient data will be monitored for completeness by independent assessors to ensure blinding of outcome measurements.

#### Outcome Assessment

Blinding of patient outcome assessment is possible with a lifestyle intervention targeted at practice level as the patient only assumes they are receiving lifestyle intervention by their family physician as part of a treatment program in a research study and is not aware of the intensity of the intervention vs. a usual care control. Further, as the assessors within the central laboratory are also blinded to the treatment, this will help to maintain the blind. We will request that individual practices not discuss the trial with colleagues and other practices in an attempt to minimize bias and contamination.

### Methodological Considerations & Limitations

The structure of primary care practice in Canada is one of a nested hierarchy with patients grouped within primary care practices, some of which are within primary care groups within local health authorities. It was inevitable within this setting that cluster-randomized control trials were used to assess our new mode of lifestyle therapy. As cluster randomized trials are becoming more common, it is worth considering some of the practicalities of such a trial design in the field of lifestyle intervention of this particular project.

The first issue identified during pilot work was that of potential contamination. Although general practitioners tend to "nest" within practices, many other health professionals and staff may move through multiple practices. This was not the case within the current research network; however, this could be an area of concern for wider intervention dissemination. If this potential contamination is ignored however this could lead to dilution of estimates of intervention effect. A second issue stems from the potential slow recruitment of patients in some clusters. While recruitment rates in this trial and previous trials in our experience have been quite good and given that recruitment has to occur prior to randomization in order to prevent bias within individual patients as opposed to practice, this could lead to a lack of incentive among some patients for participation. Further, the resulting delay between recruitment and allocation times allow for patient withdrawals and for conversion of patients to hypertension and T2D which may affect comparability among subjects. Patient withdrawal prior to allocation to intervention however will not bias results, but sample size estimates may need to be inflated to take into account the number of patients recruited who are not eligible at randomization either due to conversion to the clinical syndrome or due to simple withdrawal. Early patient recruitment has several advantages as well as several disadvantages. The large number of patients involved may be a limit to the accommodation of patients involved in clinical trials within the constraints of general practice. However, given that the current study only required 10 patients per practice, this is a relatively small-added time burden to the recruited practices and was not a key concern. It is not our experience that cluster randomized trials in primary care require any additional time or financial support as compared to single centre randomized controlled trials. In fact, since these studies tend to reflect the usual care within practice, cluster randomization seems to ideal to allowing all patients and physicians to engage in the same level of care without discrimination or necessarily added time/resource burden.

## Discussion

The health benefits and implications of the SNAC lifestyle intervention could be substantial. In patients with a high risk for hypertension and T2D, each component of the SNAC intervention, including increasing physical activity and alteration of dietary intake, have been shown to reduce blood pressure and blood glucose in previous studies. However, a lack of implementation of these interventions using tailored primary care approaches has restricted the impact. The net effect of these small improvements in clinical variables can have quite significant effects on the prevention of hypertension and T2D, and hence cardiovascular risk [46]. Hence, the effects of the SNAC intervention on cardiovascular outcomes could be impressive.

It has been suggested that a population-wide approach of the DASH dietary pattern could reduce the incidence of coronary heart disease by 15% and stroke by 27% in U.S. adults just from BP reduction alone [47]. We suggest that the net effect of SNAC could be substantiately greater due to a combined lifestyle intervention but also, since the adherence and implementation to such a program could be greater through delivery in a family practice setting this seems even more likely than was observed in previous studies [48]. Further, we will identify the underlying behavioral and physiological determinants of these clinical changes. This will improve our understanding of the global improvement in patient cardiovascular risk.

Several aspects of the SNAC trial design and intervention strategy are also noteworthy. Firstly, SNAC will testing the recommendations of several policy-making organizations for cardiovascular health. These generally include adoption of low to moderate levels of physical activity, but in this trial using an exercise prescription, the adoption of higher levels of physical exercise to improve fitness as has been recommended for optimal cardiovascular benefit will be tested [49]. We also include the specific adoption of heart-healthy Mediterranean diet that is matched to level fitness of the individual regarding changes in blood pressure, glucose, behavioural and physiological indices [42]. This has not been reported to date. The exercise and diet prescription adherence will be enhanced through behavioural lifestyle modification such that tailored support will be delivered as a component of the SNAC prescription. Secondly, few trials to date have tested the effects of a comprehensive lifestyle modification within family practice and none to our knowledge have studied a matched exercise diet prescription. This is important as family practice is identified as the optimal delivery system and preferred source for adoption and maintenance of health lifestyles in the longer term. Most previous trials have attempted to isolate the effects of specific outcomes like blood pressure or blood glucose on single factors, such as weight loss alone or physical activity alone, rather than combining factors that is certainly relevant to the patients in the primary care setting. This makes sense. While the efficacy of the DASH diet has been demonstrated in controlled studies, this has not been demonstrated in free-living individuals or a delivery system such as in primary care where optimal contact with patients at risk will be observed. Hence, SNAC offers an opportunity to determine the extent to which community living individuals in primary care can adhere to a lifestyle intervention along current recommendations.

Comparison of the SNAC intervention with the usual care control will help to give comparison of usual clinical practice towards lifestyle intervention in primary care. Hence, we will be able to test our survey data suggesting low levels of physical activity prescription are performed in primary care [16]. While a significant difference between the two arms in this study are anticipated and would provide further support for public health policy advocating adoption of healthy lifestyles including those prescribed using SNAC, a finding of no significant difference between these interventions would also provide important information. Such a result may reflect either no true difference between groups in which case improved lifestyle counseling of usual care may be very effective when observed in the research setting. An alternative view may be that there was an inability of participants to make changes as suggested in the SNAC intervention. Regardless of outcome, additional insight into the observed results will come from secondary analysis that will document the extent to which participants were able to achieve the intervention targets for clinical, behavioural, and physiological outcomes in the short as well as long-term.

An important design consideration has been the inclusion of blood pressure measurement technique using an automated device. This technique would reduce the measurement bias as well as provide standardization of technique between practices which may not have been the case in previous studies. This will be corroborated using 24-hour ambulatory technology. The inclusion of persons with above optimal, but not hypertensive blood pressure is a significant population at risk at a time when lifestyle mediated intervention can significantly reduce the blood pressure-related and glucose-related cardiovascular morbidity and mortality. Linking clinical, behavioral and physiological outcomes in this study is truly translational research at the point of care.

The results of the SNAC study could influence policy pertaining to the implementation of lifestyle recommendation in the comprehensive management of patients with early cardiovascular risk as well as those with established risk factors [17]. Despite regular, widely distributed guidelines suggesting the importance of lifestyle modifications of basis for therapy and prevention, implementation of such guidelines into routine medical practice has been suboptimal. At present, implementation relies on physicians without training, interventions or tools. This presents barriers to delivery that will be directly addressed in the current trial where physicians will be provided with validated, tailored clinical practice tools developed for their setting. This would be particularly important to contemporary health care systems such as managed care organizations, who offer specialized health education and prevention programs as well as newly implemented family health care teams in Ontario, who could potentially implement such programs as SNAC for global cardiovascular risk prevention and control. These programs are currently reimbursed by health insurance and are provided in both fee-for-service settings, as well as managed care organizations. Hence, there is a precedent for implementation of the SNAC lifestyle intervention to control and prevent cardiovascular disability.

In summary, it is anticipated that the results of the SNAC trial will provide scientific rationale in support of an evidence-based multi-component lifestyle intervention program designed to reduce the risk of hypertension and T2D at the primary care level. Translation of clinical and laboratory based physiological measures will help establish the validity of these findings as an important part of preventive practice for family physicians.

## Abbreviations

T2D: type 2 diabetes; SNAC: Staged Nutrition and Activity Counseling trial; STEP: Step Test Exercise Prescription; TIA: Transient ischemic attack; DASH: Dietary Approaches to Systolic Hypertension trial; CRP: C-reactive protein; VO2max: maximal aerobic capacity; BMI: body mass index; ICC: interpractice correlation coefficient; VCAM: vascular adhension molecule; ICAM: intracellular adhesion molecule; HNBP: high-normal blood pressure; IGT: impaired glucose tolerance; IFG: impaired fasting glucose; BP: blood pressure; ANOVA: analysis of variance; ANCOVA: analysis of covariance; BMI: Body mass index; CRP: C reactive protein; ICAM-1: Intracellular cell adhesion molecule 1; ICC: Interpractice correlation coefficient; IGT/IFG: Impaired glucose tolerance/impaired fasting glucose; T2D: Type 2 diabetes; SNAC: Staged nutrition and activity counseling trial; STEP: Step test exercise prescription; **TIA: Transient ischemic attack; VCAM: Vascular adhesion molecule; VO**_**2 max**_**: Maximal aerobic apacity or fitness**

## Competing interests

The authors declare that they do not have competing interests.

## Authors' contributions

RP-was involved in obtaining funding, the design, training, conduct, analysis and writing of this paper. KA-was involved in the conduct, analysis and writing of this paper. KS-was involved in the design, training, analysis and writing of this paper. LP-was involved in the design, training and analysis of this paper. TO-was involved in the training, analysis and writing of this paper.
